# Meta-analysis: Adefovir dipivoxil in combination with lamivudine in patients with lamivudine-resistant hepatitis B virus

**DOI:** 10.1186/1743-422X-6-163

**Published:** 2009-10-09

**Authors:** En-Qiang Chen, Li-Chun Wang, Jun Lei, Lu Xu, Hong Tang

**Affiliations:** 1 Center of Infectious Diseases, West China Hospital, Sichuan University, Chengdu, Sichuan 610041, PR China; 2 Division of Infectious Diseases, State Key Laboratory of Biotherapy, Sichuan University, Chengdu, Sichuan 610041, PR China

## Abstract

**Background:**

Currently, there are no conclusive results on the efficacy of adefovir dipivoxil (ADV) plus lamivudine (LAM) in LAM-resistant patients with chronic hepatitis B (CHB). The aim of study was to evaluate the efficacy of rescue therapy with ADV plus LAM compared to ADV monotherapy in LAM-resistant CHB patients.

**Results:**

We searched PUBMED, EMBASE, Web of Science, CNKI (National Knowledge Infrastructure), VIP database, the Cochrane Central Register of Controlled Trials and the Cochrane Database of Systematic Reviews. Six eligible trials (442 patients in total) were included and evaluated for methodologic quality and heterogeneity. Greater virological response and lower emergence rate of ADV-associated mutants was observed in ADV plus LAM compared to ADV monotherapy (both *P *< 0.05). On the contrary, the rate of ALT normalization, HBeAg clearance and seroconversion were all similar between ADV plus LAM and ADV (all *P *> 0.05). Additionally, adding-on or switch-to ADV was both well tolerated.

**Conclusion:**

The combination of ADV with LAM was superior in inhibiting HBV replication and preventing drug resistance as compared to ADV alone for LAM-resistant CHB patients.

## Background

Chronic infection with hepatitis B virus (HBV) represents a global health problem, with continuing new infections worldwide, being an important cause of liver disease, morbidity, and mortality [1,2]. The goals of therapy in HBV infected patients are to limit or reverse progression of the disease through sustained suppression of HBV replication [3,4]. Effective treatment of chronic hepatitis B (CHB) improves significantly patients' survival and reduces the risk of development of major complications[1]. Lamivudine (LAM) is the first nucleoside analog approved for the treatment of CHB, and it has been and is still widely applied globally for CHB patients. But it has the major limitation of the development of drug-resistance mutants occurring at a rate of 16%~32% during the first year of treatment and increasing by 15% with each year of additional treatment [5,6]. Available clinic data showed that the emergence of LAM-resistant mutations can be associated with hepatitis flares, hepatic decompensation and death [7,8].

Adefovir dipivoxil (ADV) as an oral prodrug of an acyclic monophosphate adenine analog, has gained popularity as a first-line treatment modality for patients with compensated hepatitis B, by virtue of its satisfactory efficacy coupled with an relatively good record of safety [9-11]. Given the complementary resistance profile of HBV with LAM and ADV, it has been regarded as a rescue therapy for LAM-resistant CHB patients either in monotherapy or in combination with LAM [12,13]. The combination of ADV with LAM also has been recommended by EASL Clinical Practice Guidelines (CPGs) if tenofovir not yet available[14]. As we know, combination therapy offers several advantages over monotherapy. Combining drugs may achieve synergistic or additive antiviral effects compared with single drug therapy. For example, more rapid achievement of an undetectable HBV DNA level may increase the rates of seroconversion of hepatitis B e antigen (HBeAg) or normalization rate of ALT[15]. However, it is still debated whether the combination of ADV with LAM could improve the biochemical, virological, or serological response rates as compared with ADV alone. Some previous studies suggested that there were no obvious improvement in ALT normalization, virological suppression or reduction in the development of ADV-resistant mutations for ADV+LAM combination compared with ADV alone [16,17]. While, some other studies have reported that in CHB patients with LAM-resistance, the combination of ADV with LAM is superior to ADV alone, as it is associated with higher virological response rate and lower viral resistance rate [18-21].

According to the new guidelines of EASL, it is necessary to do deep into exploring various new strategies including appropriate combination therapy to achieve optimal curative effect for CHB patients[14]. At present, there were no evidence-based conclusive results on efficacy (including biochemical improvement, HBV DNA suppression, HBeAg clearance and seroconversion, and the development of subsequent ADV resistance) of ADV plus LAM combination therapy versus ADV monotherapy in LAM-resistant CHB patients. In this study, we aimed to elucidate this topic using meta-analysis of data from published randomized controlled trials (RCTs). The results of this meta-analysis indicated that the therapeutic potential of combination of ADV with LAM was beneficial for treating LAM-resistant CHB patients, with lower incidence of ADV-associated mutations.

## Results

### Characteristic and Quality of Studies

We searched relevant literatures, and finally 6 randomized controlled trials (RCTs) were left for analysis which involved 442 patients in total[20,22-26], of whom 213 were included in ADV+LAM combination therapy groups and 229 were included in ADV monotherapy groups. All included trials had clearly stated inclusion and exclusion criteria. In addition, all studied populations with comparable baseline characteristics between the combination therapy and monotherapy groups. Of the 6 trials, 3 were published in English[20,22,23] and the others were published in Chinese [24-26]. The studies were of heterogeneous methodological quality (Jadad scores ranged from 2 to 5), and the detailed information of included RCTs was summarized in table. 1 and table. 2.

**Table 1:** Treatment characteristics of clinical trials included in this study

**Study**	**HBeAg status** **(+/-)**	**Strategy for therapy**	**Primary efficacy measures**	**Secondary efficacy measures**
Rapti (2007)	-	ADV 10 mg vs. ADV 10 mg + LAM 100 mg	BR, VR	ADV-R, AE
Chen (2008)	+	ADV 10 mg vs. ADV 10 mg + LAM 100 mg	BR, VR, eAg-C, eAg-SC	No
Yang (2008)	+/-	ADV 10 mg vs. ADV 10 mg + LAM 100 mg	BR, VR, eAg-C, eAg-SC	ADV-R, AE
Peters (2004)	+	ADV 10 mg vs. ADV 10 mg + LAM 100 mg	BR, VR, eAg-C, eAg-SC	ADV-R, AE
Xiao (2008)	+	ADV 10 mg (with or without an overlap period with LAM) vs. ADV 10 mg + LAM 100 mg	BR, VR, eAg-C, eAg-SC	No
Ijaz (2008)	-	ADV 10 mg vs. ADV 10 mg + LAM 100 mg	VR	ADV-R

ADV, adefovir dipivoxil; LAM, lamivudine; eAg-C, hepatitis B e antigen (HBeAg) clearance; eAg-SC, HBeAg seroconversion; ADV-R, ADV resistance; BR, biochemical response; VR, virologic response; AE, adverse event.

**Table 2:** Characteristics of included clinical trials in meta-analysis

**Study**	**Study design**	**Jadad score**	**Sample size (monotherapy/combination therapy)**	**Duration of treatment** **(week)**	**Follow-up period** **(weeks)**
Rapti (2007)	RCT	4	14/28	120/160	40
Chen (2008)	RCT	4	34/34	48	12
Yang (2008)	RCT	2	73/73	48	12
Peters (2004)	RCT, DB	5	19/20	48	12
Xiao (2008)	RCT	4	85/54	88	22
Ijaz (2008)	RCT,	3	4/4	79/71	20/17

RCT, randomized controlled trial; DB, double blind.

### Biochemical response

**Figure 1:**
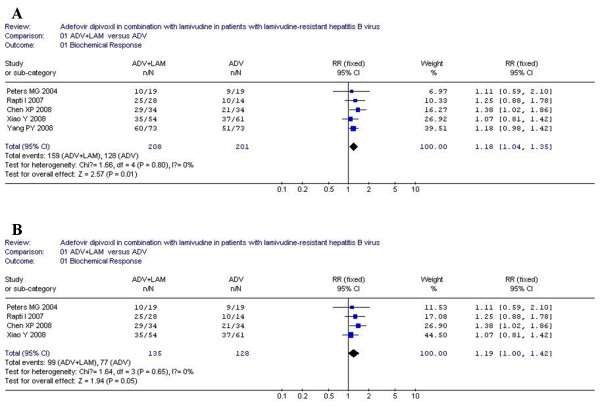
**Analysis of the biochemical response of ADV+LAM combination therapy versus ADV monotherpy for treatment of LAM-resistance CHB patients**. A: five trials were analyzed; B: four trials were analyzed.

According to chi-squared statistic and I square (I^2^), heterogeneity was assessed and not found to be a concern. Of the six included trials, only five trials demonstrated the biochemical response rate, and the biochemical response rates in ADV+LAM combination group was higher as compared with that in ADV monotherapy [159/208 vs. 128/201, RR = 1.18, 95%CI (1.04-1.35), P = 0.01](Figure 1A). However, when low-quality study was removed, the difference in response rate between two groups became similar [99/135 vs. 77/128, RR = 1.19, 95%CI (1.00-1.42), P = 0.05] (Figure 1B).

### Virological response

**Figure 2:**
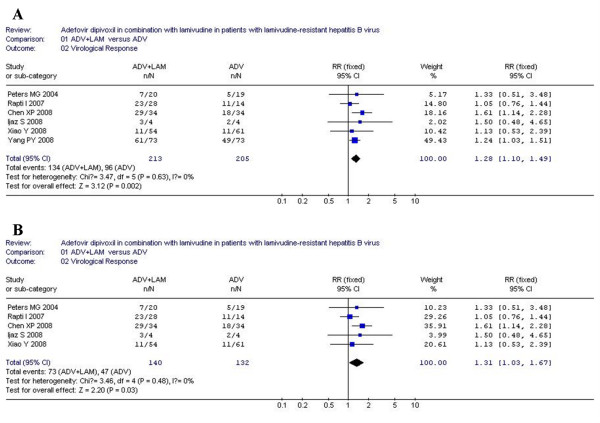
**Analysis of the virological response of ADV+LAM combination therapy versus ADV monotherpy for treatment of LAM-resistance CHB patients**. A: six trials were analyzed; B: five trials were analyzed.

According to chi-squared statistic and I square (I^2^), heterogeneity was assessed and not found to be a concern. Greater virological response rates were observed in ADV+LAM combination group as compared with that in ADV monotherapy, and the difference in response rate between two groups were statistically significantly [134/213 vs. 96/205, RR = 1.28, 95%CI (1.10-1.49), P = 0.002](Figure 2A). Additionally, when low-quality study was removed, the difference in response rate was still statistically significantly [73/140 vs. 47/132, RR = 1.31, 95%CI (1.03-1.67), P = 0.03] (Figure 2B).

### Hepatitis B e antigen clearance

**Figure 3:**
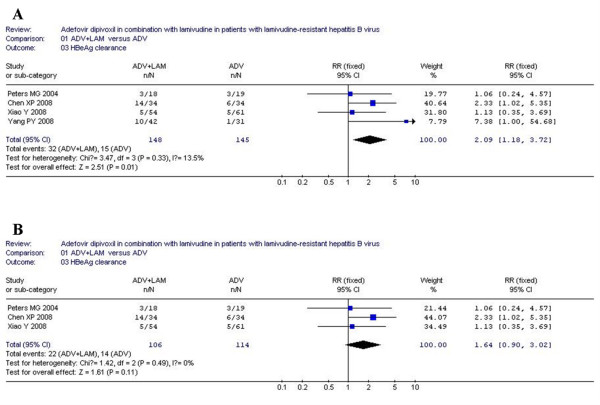
**Analysis of the HBeAg clearance of ADV+LAM combination therapy versus ADV monotherpy for treatment of LAM-resistance CHB patients**. A: four trials were analyzed; B: three trials were analyzed.

According to chi-squared statistic and I square (I^2^), heterogeneity was assessed and not found to be a concern. Of the four analyzed trials, greater HBeAg clearance was observed in combination group as compared with monotherapy [21/148 vs. 8/145, RR = 2.47, 95%CI (1.15-5.28), P = 0.02](Figure 3A). However, when low-quality study was removed, the difference in HBeAg clearance between two groups became similar [14/106 vs. 7/114, RR = 2.02, 95%CI (0.89-4.57), P = 0.09] (Figure 3B).

### Hepatitis B e antigen seroconversion

**Figure 4:**
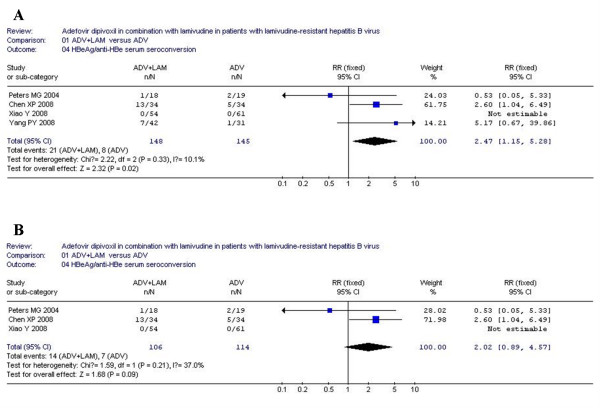
**Analysis of the HBeAg/anti-HBe serum seroconversion of ADV+LAM combination therapy versus ADV monotherpy for treatment of LAM-resistance CHB patients**. A: four trials were analyzed; B: three trials were analyzed.

According to chi-squared statistic and I square (I^2^), heterogeneity was assessed and not found to be a concern. Of the four analyzed trials, greater HBeAg seroconversion was observed in combination group as compared with monotherapy [21/148 *vs*. 8/145, RR = 2.47, 95%CI (1.15-5.28), P = 0.02](Figure 4A). However, when low-quality study was removed, the difference between two groups became similar [14/106 *vs*. 7/114, RR = 2.02, 95%CI (0.89-4.57), P = 0.09](Figure 4B).

### Emergence of ADV-resistant mutation

**Figure 5:**
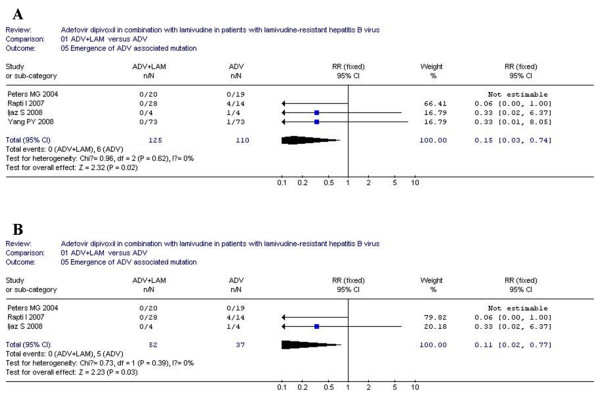
**Analysis of the ADV-resistance mutation of ADV+LAM combination therapy versus ADV monotherpy for treatment of LAM-resistance CHB patients**. A: four trials were analyzed; B: three trials were analyzed.

Of the six included studies, four studies detected ADV-resistant mutants during the course of treatment. According to chi-squared statistic and I square (I^2^), heterogeneity was assessed and not found to be a concern. Greater emergence rates of ADV-resistant mutants were observed in ADV monotherapy as compared with ADV+LAM combination therapy [0/125 vs. 6/110., RR = 0.15, 95%CI(0.03-0.74), P = 0.02](Figure 5A). Interesting, after low-quality study was removed, the difference in ADV-associated mutations was still statistic significantly between two group [14/106 vs. 7/114, RR = 2.02, 95%CI (0.89-4.57), P = 0.09](Figure 5B).

### Safety

For majority of the included trials, long-term ADV treatment in LAM-resistance chronic hepatitis B patients either alone or in combination with LAM, was general well tolerated. Only Rapti I *et al*.[22] in their study reported that 3 patients with cirrhosis in ADV+LAM combination therapy developed HCC, and 2 patients with cirrhosis had a decrease of creatinine clearance in combination therapy group, but no statistically significant difference were seen between combination of ADV with LAM and ADV alone.

## Discussion

The prevalence of drug-resistant mutants in patients is associated with the loss of clinical and virological benefits[27], and may limit future therapeutic options. So prevention is important for long-term therapeutic efficacy. The use of combination therapy is particularly important given the limited number of treatment options available for patients with drug-resistant HBV infection. In successful antiviral therapy of patients, drug combinations can delay or prevent the emergence of drug-resistant mutants. However, combination therapy was recommended only when drugs with a low barrier to resistance are used, such as LAM and ADV. And combined drugs should have complementary resistance profiles[15]. Recently, there are a growing number of studies of ADV and LAM combination therapy in treatment-experienced patients with LAM-resistant HBV, and their results indicated that the combination of ADV with LAM is superior to ADV alone in patients with LAM-resistant HBV infection[18,20,28]. However, the overall benefits of the combination of ADV with LAM have not been fully assessed quantitatively. This study is the first to examine the combination of ADV with LAM for LAM-resistant patients, pooling data from related trials into meta-analysis, and the results of this study will aid in achieving evidence-based conclusions on the advantages of combination therapy of ADV with LAM.

As we know, LAM-resistant and wild-type HBV likely coexist as vial quasispecies in patients. Because either ADV or LAM exert antiviral activity against wild-type HBV, and LAM-resistant HBV are still hypersensitive to ADV, the combination of LAM and ADV could inhibit the replication of wild-type and LAM-resistant HBV collaboratively[29]. In this meta-analysis, ADV administered in combination with LAM was found to be effective, associated with greater virological responses rates and lower drug resistant rates as compared to ADV alone in patients with drug-resistant to LAM. Despite the limited number of patients and relatively short duration in two groups, the difference in virological response rate is still statistically significant (P < 0.05). Results from currently available studies of ADV therapy in patients with clinical resistance to LAM suggest an inverse relationship between pretreatment levels of viremia and antiviral response[6]. The power of pretreatment viremia in predicting a response to ADV was supported in previous studies by the significant response to ADV in LAM-naive patients with chronic HBV infection and circulating low pretreatment levels of HBV DNA. In this meta-analysis, there was no evidence of pre-existing ADV-resistant variants, which may be correlated with the significant virological response in a short period[30], especially for patients receiving combination of ADV with LAM. Moreover, longer duration treatment showed superiority of combination therapy over ADV alone in preventing ADV resistance [20], which is consistent with the results of this study. For example, the results of a retrospective-prospective study consisting of 585 patients with LAM-resistant CHB treated with combination of ADV with LAM or ADV alone for a median of 33 months suggested that the 3-year cumulative risk of ADV resistance was 16% in the ADV alone versus 0% in the combination of ADV with LAM (P < 0.001)[31].

It has been identified that LAM-resistance mutations occurs as a result of mutation at position 204 or 180 of the HBV DNA polymerase, which remain sensitive to ADV. Resistance to ADV is prevalent due to rtA181V and rtN236T mutations[29]. Recently, someone reported that a single amino acid change at position rt181 may induce cross-resistance to LAM and ADV[32], but this phenomenon was not widely observed in clinical practice. In another word, majority of currently available data suggested that the combination of ADV with LAM could eliminate or hamper the emergence of ADV-resistant strains for LAM-resistant patients. So, the possible explanation for this low resistant rate is the ability of ADV and LAM in combination to cross-inhibit the corresponding drug-related HBV mutants, thereby preventing accumulation of mutated strains with adequate fitness for replication. However, the combination of ADV with LAM did not completely prevent virological breakthrough or drug resistance in all patients.

In this meta-analysis, either combination of ADV with LAM or ADV alone was well tolerated. And the emergence rate of adverse events was not significant to combination as compared to ADV alone, even limited data were available regarding renal toxicity when safety was assessed. In one study of 145 patients with LAM resistance, only 8% of patients developed mild nephrotoxicity, but all were able to continue combination therapy after increasing the ADV-dosing interval[33]. And in another RCT studies of LAM versus LAM plus ADV including 115 treatment-native patients, none of the patients receiving combination therapy developed nephrotoxicity[34].

Recently, combination of ADV with LAM was also recommended as a good choice for LAM-resistant patients by the new guidelines of American Association for the Study of Liver Diseases (AASLD) and Asian Pacific Association for the Study of Liver (APASL). After referring to the results of this meta-analysis and wealth of data presented by previous retrospective and prospective trials of larger sample, we believe that the combination of ADV with LAM would be a good option for LAM-resistant patients.

## Conclusion

The combination of ADV with LAM was superior in inhibiting HBV replication and preventing drug resistance as compared to ADV alone for LAM-resistant CHB patients. It is thus recommended that the combination therapy of LAM plus ADV could be used as an option for patients with LAM-resistant HBV infection.

## Methods

### Search strategy

We searched the following databases until February 2009: PUBMED from 1966, EMBASE from 1966, Web of Science from 1955, CNKI (National Knowledge Infrastructure) from 1980, and Chinese VIP database from 1989. The Cochrane Central Register of Controlled Trials and the Cochrane Database of Systematic Reviews were also searched. Of these databases, CNKI and VIP databases provide literatures in Chinese. In this study, the search was designed using "adefovir", "lamivudine", "lamivudine failure", or "lamivudine resistance". Reference lists from retrieved documents were also searched. To maximize data requisition, we contacted authors whose articles contained inadequate information.

### Inclusion and exclusion criteria

The following inclusion criteria were used: (i) study design: randomized controlled trial, (ii) study population: LAM-resistant CHB patients; (iii) intervention: ADV monotherapy versus LAM and ADV combination therapy. Our search was limited to human studies and the following exclusion criteria were used: (i) examining the nonadult population; (ii) not reporting any of the primary efficacy measures as defined by the authors.

### Data extraction

Two investigators independently screened titles and abstracts, selected the trials and performed the data extraction. The conflict in data extraction was resolved by discussion among investigators and reference to the original article. In some cases, original principal investigators were contacted to collect information that was collected but not published. When several publications pertaining to a single study were identified, the most recent and complete publication was used.

### Efficacy measures and definitions

The rates of biochemical response, virological response, and HBeAg seroconversion were used as primary efficacy measures. Emergence of ADV associated mutation and the safety of combination therapy were used as secondary efficacy measures. Biochemical response was defined as normalization of ALT levels; Virological response was defined as attainment of undetectable levels of HBV DNA; HBeAg clearance was defined as HBeAg disappearance and seroconversion was defined as HBeAg antibodies appearance. The emergence of ADV resistance was defined as the detection of rtN236T or rtA181V mutation during the follow-up period and the safety of treatment was assessed using the occurrence rate of adverse events including renal dysfunction, decompensation of cirrhosis and hepatocellular carcinoma (HCC).

### Study quality

The methodological quality of included trial was assessed using the modified Jadad quality scale, which was calculated by assessing four items: random sequence generation, allocation concealment, blind method, and description of withdrawals and dropouts. The qualities of the first three items were classified into three grades respectively: adequate (2 points), unclear (1 point), and inadequate (0 point). And the fourth item was classified into two grades: described (1 point) and not described (0 point). The scores of modified Jadad quality scale were ranged from 0 to 7, with scores ≥ 4 signifying high-quality studies. Heterogeneity was assessed for each analysis.

### Data analysis

Data analysis was carried out with the use of Review Manager Software 4.2 (Cochrane Collaboration, Oxford, United Kingdom). For each eligible study, dichotomous data were presented as relative risk (RR), and continuous outcomes were presented as weighted mean difference (WMD), both with 95% confidence intervals (CI). Meta-analysis was performed using fixed-effect or random-effect methods, depending on the absence or presence of significant heterogeneity. Statistical heterogeneity between trials was evaluated by the chi-square and I-square (I^2^) tests, with significance set at *P *< 0.10. In the absence of statistically significant heterogeneity, the fixed-effect method was used to combine the results. When heterogeneity was confirmed (*P *< 0.10), the random-effect method was used. Additionally, sensitivity analysis should be carried out if low quality trials were included. The overall effect was tested using *z *scores, with significance set at *P *< 0.05.

## Competing interests

The funding source had no influence on study design, in the collection, analysis, and interpretation of the data, in the writing of the manuscript, or in the decision to submit the manuscript for publication. The contents are solely the responsibility of the authors and do not necessarily represent the views of the funding source.

## Authors' contributions

TH conceived the study, provided fund supporting and revised the manuscript critically for important intellectual content. CEQ and WLC made substantial contributions to its design, acquisition, analysis and interpretation of data. LJ and XL participated in the design, acquisition, analysis and interpretation of data. All authors contributed equally to this manuscript. All authors read and approved the final manuscript.
